# Cervical Thymic Cyst Mimicking Laryngocele

**DOI:** 10.1155/2013/839406

**Published:** 2013-12-17

**Authors:** Kayhan Ozturk, Cagdas Elsurer, Serap Bulut, Mutlu Duran, Serdar Ugras

**Affiliations:** ^1^Department of Otolaryngology Head and Neck Surgery, SelÇuk University Faculty of Medicine, SelÇuk University, Alaeddin Keykubat Campus, 42075 Selcuklu, Konya, Turkey; ^2^Department of Pathology, SelÇuk University Faculty of Medicine, SelÇuk University, Konya, Turkey

## Abstract

Cervical thymic cysts are nearly 0.3% of all congenital cervical cysts. Thymic cysts are asymptomatic, but they rarely complain of dysphagia or tracheal obstruction symptoms. A soft, mobile, and painless mass increasing with valsalva maneuver directs the diagnosis of laryngocele. There has not been any study in the literature in which thymic cyst presenting like laryngocele. We hereby present a case of thymic cyst mimicking laryngocele that has not been reported so far.

## 1. Introduction

Thymic cysts are rare lesions and may occur anywhere from mandible to mediastinum, along with the migration path of the thymus. However, they are mostly seen in the mediastinum. Thymic cysts are generally misdiagnosed as branchial cyst and cystic hygroma, since they may cause almost similar symptoms to cervical cysts. However, a thymic cyst mimicking a laryngocele is extremely unusual. We herein present a boy with a cervical thymic cyst mimicking laryngocele. To the best of our knowledge, this is the first case in the literature showing that a cervical thymic cyst presenting as a laryngocele in childhood age.

## 2. Case Report 

**Figure 1:**
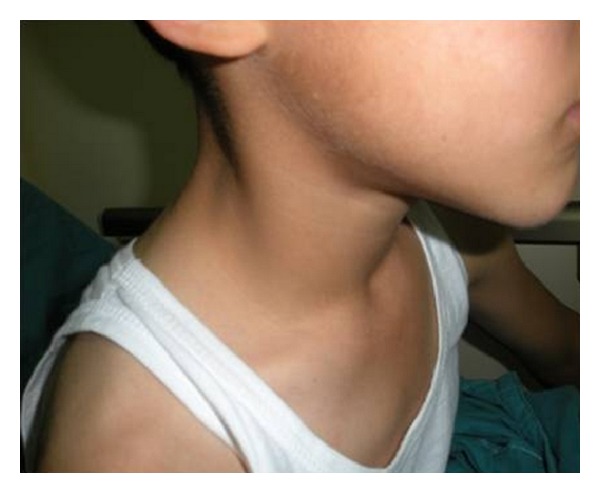
A 2 cm painless mass on the right side of the neck in a 7-year-old patient.

**Figure 2:**
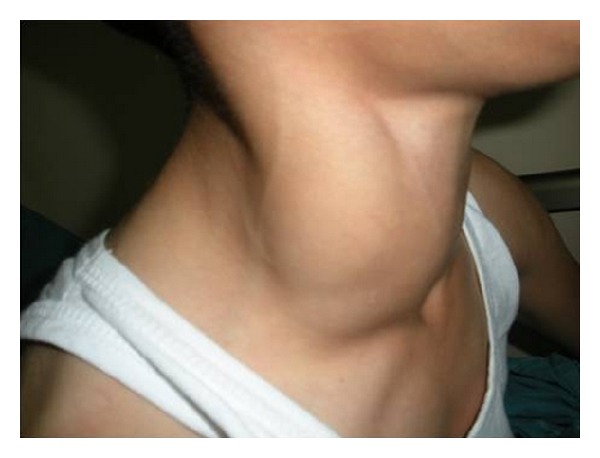
The mass increased in size and became visible with the valsalva maneuver.

**Figure 3:**
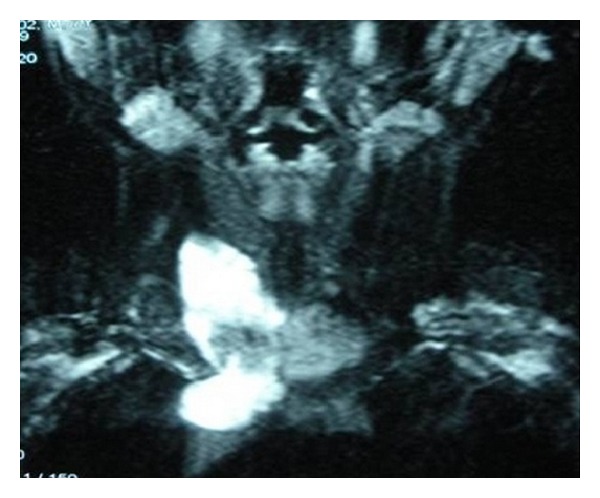
Magnetic resonance imaging of the cervical cyst disclosing its relation to the larynx.

**Figure 4:**
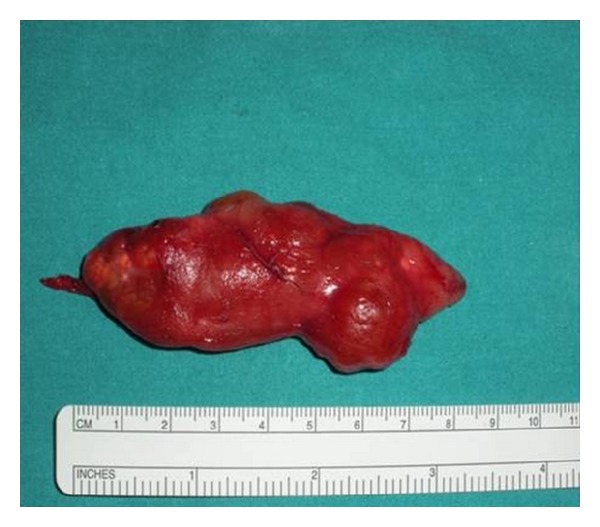
The gross appearance of the dissected cervical mass and the surrounding tissues.

**Figure 5:**
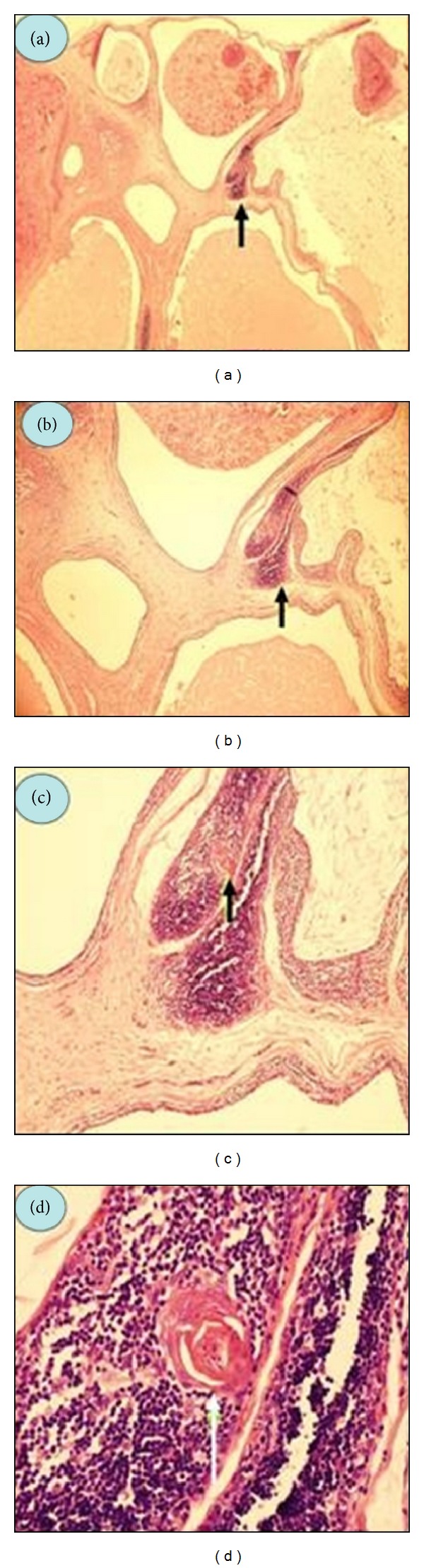
(a) Multilocular cyst and lymphoid tissue (arrow) (Hematoxylin-eosin stain, original magnification, ×20); (b) Lymphoid tissue (arrow) in the cyst wall (Hematoxylin-eosin stain, original magnification, ×40); (c) Hassall's corpuscle within the lymphoid tissue (Hematoxylin-eosin stain, original magnification, ×100), (d) Hassall's corpuscle (Hematoxylin-eosin stain, original magnification, ×400).

A 7-year-old patient admitted to our clinic with a painless mass on the right side of the neck, which was 2 cm in diameter (Figure 1). The detailed anamnesis revealed that the swelling occurred suddenly a few years ago and progressively increased in size thereafter. The patient did not have any other symptoms except for the sudden swelling. Physical examination disclosed a soft, mobile, and painless mass on the right side of the neck. With valsalva maneuver, it was observed that the mass increased in size and became visible (Figure 2). The patient did not complain of dysphagia, stridor, or dyspnea. Systemic physical examination was within normal limits. A successive computed tomography (CT) scan and magnetic resonance imaging (MRI) was performed which disclosed a cystic mass extending into the right hemithorax. Additionally in MRI, the cyst was in relation to the larynx (Figure 3). Based on the aforementioned clinical and radiological examinations, the patient was operated on with a preoperative diagnosis of a laryngocele. During the surgery, a 6 cm long horizontal skin incision beginning from the 4 cm superior of the right clavicle was performed. The cystic mass was made visible by dissecting the sternocleidomastoid muscle laterally and the platysma. The cervical part of the mass was dissected from the surrounding structures. It was seen that the mass was in very close proximity to the pyriform sinus, jugular vein, and internal carotid artery. Moreover, the inferior part of the mass extended to the right hemithorax towards the apex of the lung and was not attached to the pleura. The mass was dissected from the surrounding tissues and totally excised (Figure 4). No postoperative complication was observed in the patient and he was discharged on the fifth postoperative day. In the macroscopical examination, the mass was 8 × 2,5 × 1,5 cm in size and there was a dark yellow jelly material within the cyst. The dissection material also included lymphoid tissue, the largest of which was 2 × 1 × 0,8 cm in diameter and the smallest of which was 0,5 cm in diameter. The pathological diagnosis was compatible with the multilocular thymic cyst as thymus tissue and a cholesterol granule (Figure 5).

## 3. Discussion

The thymus gland is the central organ of the lymphoid system during infancy. It embryologically develops from the third and the fourth pharyngeal arches in the 6th gestational week of development. During the 7th and 10th weeks, as the primordial thymus migrates caudally and medially into the mediastinum, the proximal portion of the thymopharyngial duct usually undergoes atrophy. In the children, cervical thymic cysts constitute 0.3% of all congenital cervical cysts. Thymic cysts are rare lesions and there are less than 100 cases, together with histopathological diagnosis-reported in the literature [1]. Sixty to seventy percent of the thymic cysts occur on the left, whereas 20–30% of them occur on the right side of the neck. Approximately, 5 to 7% of the thymic cysts occur in the midline [2, 3]. Thymic cysts are seen more frequently in men than women [1]. Our case is a rare case, in which the thymic cyst occurred on the right side of the neck in a 7-year-old boy. Although thymic cysts may occur anywhere from mandible to sternum, nearly 50% of cervical thymic cysts reach the mediastinum. The thymic cysts are seen in the period when the thymus develops fastest in the first decade, beginning from 2 years of age. They can be congenital or acquired. There are two accepted hypotheses for the pathophysiology of thymic cysts: (i) persistence of the embryonic thymopharyngeal duct tract and (ii) degeneration of the Hassal's corpuscles [3, 4].

In general, the patients with thymic cysts are asymptomatic, but they rarely complain of dysphagia or tracheal obstruction symptoms. The clinical differential diagnosis is not easy and they are often misdiagnosed as cystic hygromas or branchial cleft cysts, which are also frequently seen in this age group and which have similar symptoms [4–7]. Additionally, in the childhood, thyroglossal ductus cysts, cervical lymphadenopathy, benign tumors (dermoid, epidermoid, hemangioma, and lymphangioma), and malignant tumors (lymphoproliferative diseases, soft tissue sarcomas, and other metastatic lesions) should be considered in the differential diagnosis of the a cervical thymic cyst [3, 5]. Definitive diagnosis is done by postoperative histopathological examination.

In the present case, the size of the mass increased with valsalva maneuver. Besides, there was a relationship between larynx and the mass, like a tail sign. The mass extended to the right hemithorax and there was a relationship between the mass and pleura. Our hypothesis is that during resting, the mass was located into the thorax while during valsalva maneuver, the pleura pushed the mass to the neck and the mass seemed to inflate by breath. Therefore, preoperative diagnosis of the present case was a giant external laryngocele extending into the mediastinum. We performed total excision of the mass with surgery. The excision of the lesion was curative and no reoccurrence was reported. Long-term prognosis was perfect. Our patient was followed for 16 months without recurrence. To the very best of our knowledge, this is the first case of a cervical thymic cyst mimicking laryngocele in childhood age.

In conclusion, although extremely rare, cervical thymic cysts should be considered in the differential diagnosis of the cases with the external laryngocele, particularly in the childhood age. The definitive method of treatment of the cervical thymic cyst is surgery.
